# Analysis of the growth performance, stress, profile of fatty acids and amino acids and cortisol in Tilapia (*Oreochromis niloticus*), cultured at high stocking density using in-pond raceway system

**DOI:** 10.1016/j.sjbs.2021.08.048

**Published:** 2021-08-23

**Authors:** Shafaq Fatima, Wajeeha Komal, Farkhanda Manzoor, Asma Abdul Latif, Razia Liaqat, Shahida Ameen, Raja Shahnawaz Janjua

**Affiliations:** a Lahore College For Women University, Pakistan; b SoyPak, Pvt. Ltd, Pakistan

**Keywords:** In-pond raceway system, Tilapia, High stocking density, Production, Nutritional quality

## Abstract

In-pond raceway system technology (IPRS) was introduced in Pakistan in 2019 as solution for sustainable aquaculture approach by effectively increasing production, reducing pollution and facilitating feed and pond management. Fingerlings of GIFT Tilapia (*Oreochromis niloticus*) (n = 16,500 in each raceway, initial weight = 32.00 ± 1.26 g) were stocked in June 2019 in two IPRS raceways (area of each raceway = 220 m^3^) for 171 days until harvested on November 30, 2019. Fingerlings stocked in traditional earthen ponds (area of each pond = 6167 m^3^) were studied as control (n = 3000 in each pond, initial weight = 32.00 ± 1.26 g). Average harvested biomass from raceways was 57.33 kg/m^3^ with an average daily weight gain per fish of 4.47 g per day. On the other hand, average harvested biomass from control ponds was 0.38 kg/m^3^ with an average daily weight gain per fish of 4.60 g per day. Average feed conversion ratio (FCR) in both raceways and control ponds was recorded as 1.25 and 1.24, respectively. Overall survival rate in both raceways and control ponds was above 99%. No sign of any disease was noted at any stage in both study groups. Crude protein and fats contents did not reduce in any raceway despite of high stocking density and sharp seasonal changes. Profile of essential and non-essential amino acids were found to be upto nutritional requirements of adult human. High levels of n-3 and n-6 fatty acids in fish collected from raceways as compared to those in traditional earthen pond show that muscle quality was not compromised due to high stocking density in small area. Return on investment excluding capital cost was 47.05 which implies that IPRS technology can be economically feasible with further modifications.

## 1 Introduction

With expansion of global aquaculture production (179 million tonnes in 2018), there is constant threat towards food safety and sustainability of water and land resources (FAOSTATJ, 2019). In-pond raceway system (IPRS) technology has gained attention during last few years as an economic and environmentally sustainable aquaculture production system proved to be successful for channel catfish, hybrid catfish, grass carp, tilapia and largemouth bass (Brown et al., 2011, Arana et al., 2018, Wang et al., 2019, Yuan et al., 2019). Floating and fixed raceways system had successfully resolved the problem of consistent standard aeration, removal of solid waste in one integrated and more controllable system than traditional pond culture (Masser, 2004, Brown et al., 2011, Chappell, 2017).

In Pakistan, cyprinids (major carps) (153,937 MT) and Tilapia (245 MT) farming contribute the most in total annual freshwater aquaculture production (200,003 MT) (FAOSTATJ, 2019). Total land area used for fish farming is 60,470 hectare (ha). Per capita consumption of fish is 1.90 kg which is expected to be up to 5–6 kg by 2025 considering the projected increase in population (FAOSTAT, 2019). To fulfill the demand of increasing population, more land and water resources will be required to build fish farms. However, Pakistan's per capita annual water availability is 1017 m^3^ which is perilously close to the water scarcity threshold of 1000 m^3^ (UNDP, 2016). Similarly, cost of land or lease are too high to efficiently support further development. Moreover, sustainability of natural water and land reserves will be compromised as no strict legislation operates in aquaculture sector regarding environment protection in Pakistan.

The traditional pond system typically produces 2600 kg/ha of fish recording average FCR of 5–7 while mostly using powdered feed with limited feed ingredients (Janjua, 2015). Neither dissolved oxygen is maintained above standard value of 3 mg/L (Boyd and Hanson, 2010) nor better feeding management is practiced. Considering the future challenges to meet animal protein demand, there is urgent need to improve traditional farming methods, demanding more efficient production technologies to intensify culture but maintain water quality and sustain the water and land reserves simultaneously. Cage culture and recirculation aquaculture systems (RAS) have not proved to be economically feasible in Pakistan to intensify fish culture.

Intensive aquaculture technologies, ensure the complete utilization of space in a production system, improve the fish production and profitability by stocking high number of fish per cubic area. Fish growth and stocking density are negatively correlated owing to the competition for food and space resulting in stress causing the growth reduction and compromising immunity (Rebl et al., 2017). Cost effective production should ensure maximum production with minimum physiological stress and disease incidence (Liu et al., 2018). Levels of cortisol is major indicator of stress. Stress induced high levels of cortisol might remarkably reduce growth by altering activity of metabolic enzymes such as hepatic aminotransferase, lipase and trypsin (Bolasina et al., 2006) and interfere with thyroid function in in certain species such as Nile tilapia (Walpita et al., 2007).

**Fig. 1:**
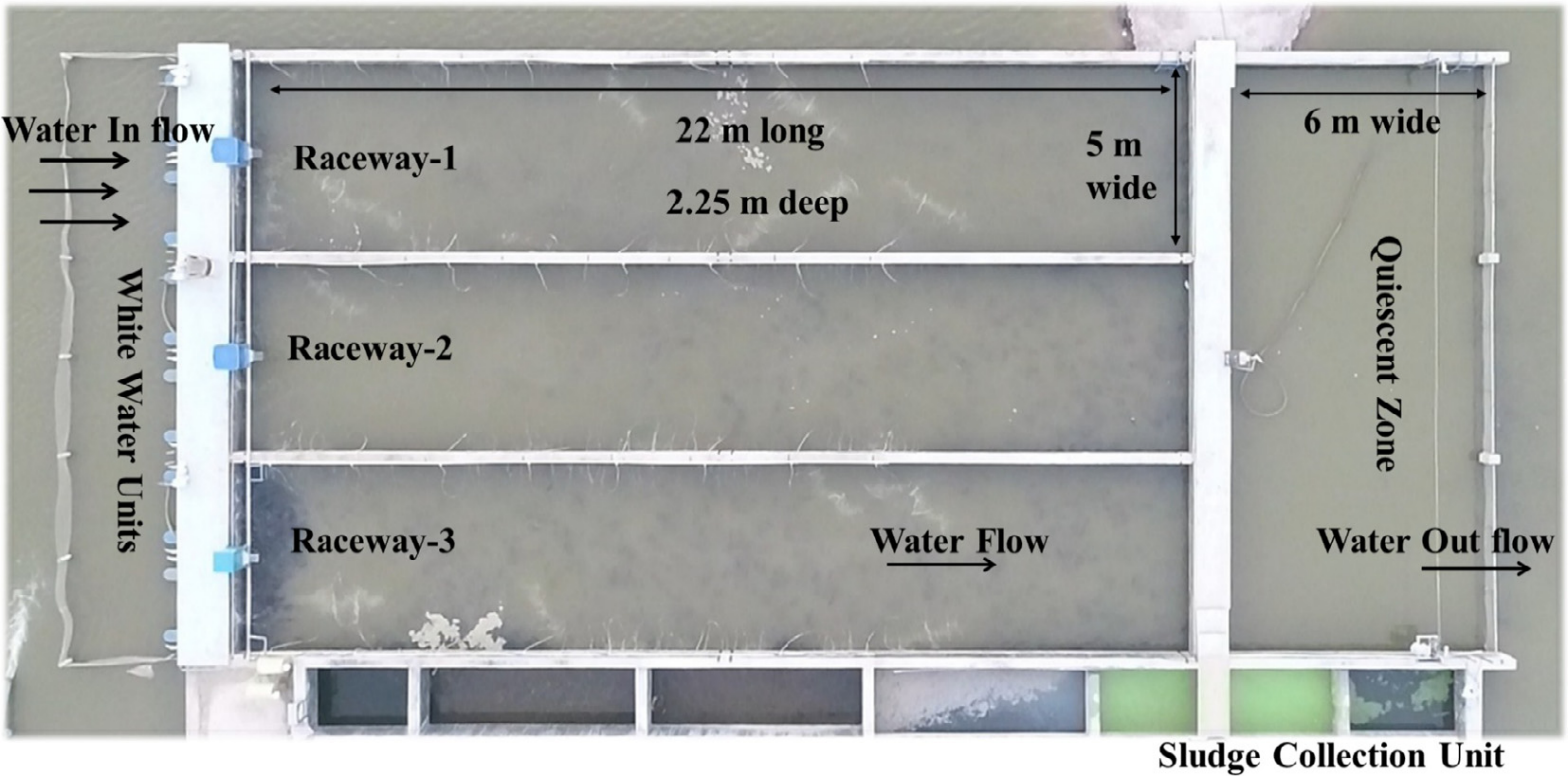
Aerial view of IPRS production system used in present study. Raceway-1 and Raceway-2 were used for the study. Dimensions of raceway are given in top raceway in the image. Diffuser grids of White Water Units are under the water. Only pontoons supporting the grids are visible. Auto feeders are fixed at each raceway. Arrows show the direction of water flow from White Water Units towards Quiescent Zone. Sludge collection unit consisted of seven compartments. Waste water was drained from these compartments through underground drain.

In-Pond raceways system was established in present study following Chappell (2017) as an in-service experience to investigate the effects of high stocking density on growth, production, flesh quality, occurrence of any disease, economic benefits and commercial feasibility of this technology in Pakistan in comparison to traditional earthen pond farming. Air lift and bottom aeration were maintained as reported by Chappell (2017), however, waste collection gear was simplified by using only one vacuum head (wiper blade) fitted with air lift pump (Fig. 1). Efficiency of this system having high stocking density of cultured species was studied in terms of water quality, fish growth, proximate composition, profile of amino acids, fatty acids and cortisol, biomass production and economic feasibility. Present study is expected to provide baseline data to further improve this husbandry of species in IPRS production system.

## 2 Materials and methods

### 2.1 IPRS system

IPRS system was constructed in earthen pond of seven acres water area (2.83 ha), supplied with well water (pH: 7.80, TDS: 352 ppm) at a commercial fish farm in east-central Pakistan (30.6432°N, 72.0201°E). IPRS system consisted of three individual raceways, constructed following Chappell (2017) (Fig. 1). Land area of each raceway (22 m long × 5 m wide × 2.25 m deep) was connected to common Quiescent Zone (QZ) (3 m long × 15.61 m wide × 2.35 m deep) and sludge collection unit (22 m long × 2 m wide × 2.5 m deep). Fish were confined in the raceway by an end partition barrier that spanned the width of each unit.

Five meter long airlift system containing, four diffuser grid racks (each 1.2 m long, 1.05 m wide) and deflection hood for each raceway were imported (XuanCheng Dingxing Environmental Protection Engineering Co., Ltd, China) and assembled as described by Chappell (2017). Aeration for each raceway was supplied by a 2.2 kW Ring blower (50 hz, 345–415 V, 19 Kpa compressor) (XuanCheng Dingxing Environmental Protection Engineering Co., Ltd, China) capable of delivering maximum airflow of 210 m^3^/h directly into the water column via two diffuser grid airlift. Each diffuser grid was built by using the anti-microbial tubing (outer diameter: 25.4 mm; inner diameter: 12.7 mm; airflow: 2.2 m^3^/h/meter). Suggested maximum airflow of one diffuser grid is 44 m^3^/h while two grids in each raceway can produce airflow of 88 m^3^/h. Bottom aeration in each raceway was also provided by 3 kW Ring blower (50 hz, 345–415 V, 41 Kpa compressor, 230 m^3^/hour airflow) and 16 pieces of anti-microbial tubing (length of each piece: 1 m long; airflow: 2.2 m^3^/h). Earthen baffle wall attached to the outside wall of third IPRS raceway extended 120 m into the pond to assist in directing the flow of water around the pond and prevent short circuiting of return flow to the raceways. Three paddle wheel aerators (1.5 kW) was placed in open pond to generate a riverine flow in open pond area to bring the water back towards the white water unit (WWU).

In QZ, a continually oscillating vacuum head (2.7 m long) were connected to collect the waste from bottom of QZ and transferred them to waste collection units. Automatic feed delivery system was installed for each raceway, each having storage capacity of 100 kg (3–20 m feed casting distance, 16 W vibration motor and 150 kg/hr maximum feeding capacity). Two generators (20 kW and 30 kW) were installed as power back up in case of power failure to run the whole system including aeration and feeding. There was zero water discharge throughout the grow out period, only the water lost by evaporation was replenished when needed. System and data maintenance activities such as water quality data collection (daily); cleaning probe membranes (daily); calibration of probes (monthly); mortality check and morts removal (daily); feed delivery system cleaning (biweekly); were carried out on a regular basis.

### 2.2 Culture conditions

Fingerlings of tilapia were procured from a local hatchery (Tawakkal Hatchery, Pakistan) (initial weight = 32.00 ± 1.26 g) were stocked in two raceways (water area of each raceway = 220 m^3^) as replicates (Table 1) on 12 June, 2019. A total of 16,500 fingerlings were stocked in each raceway. Fingerlings were also stocked in two earthen ponds (water area = 6167 m^3^) to be studied as control. However, stocking density in control ponds could not be same as in raceways (n = 16,500 or 75 fish/m^3^) as it would cause high mortality and capital loss to farmer due to lack of continuous aeration and bad water quality. Therefore, a total of 3000 fingerlings (0.49 fish/m^3^) were stocked in each control pond (available at commercial farm site) as commonly practiced in traditional faming in Pakistan. Fish were reared in IPRS raceways and control ponds from 12 June till 30 November, 2019 (171 days).

**Table 1:** Monthly variations (Mean ± SE) in water temperature, pH and dissolved oxygen in control and IPRS raceways, measured at four different times in 24 h between June till November 2019. Mean value of each parameter, measured at depth of 0.5 ft and 6 ft noted at four different times over 24 h has been presented. Monthly values of water parameters are not given for control ponds as values of temperature and pH were similar to those in raceways except dissolved oxygen. Therefore, only the range of values over the study period has been provided for control ponds.

Time	Control	June	July	August	September	October	November
**Water Temperature (°C)**							
08:00 AM	33.34 ± 0.46–21.51 ± 0.12	33.77 ± 0.15	33.44 ± 0.32	32.76 ± 0.25	31.92 ± 0.31	27.00 ± 0.32	20.61 ± 0.26
12:00 PM	33.86 ± 0.25–21.33 ± 0.23	33.82 ± 0.41	33.15 ± 0.18	33.95 ± 0.41	32.83 ± 0.41	27.50 ± 0.28	21.20 ± 0.30
04:00 PM	33.85 ± 0.32–21.53 ± 0.45	33.87 ± 0.43	32.88 ± 0.12	33.77 ± 0.42	32.92 ± 0.42	27.93 ± 0.28	21.50 ± 0.30
12:00 AM	32.68 ± 0.21–21.55 ± 0.67	33.94 ± 0.22	32.58 ± 0.45	32.48 ± 0.21	32.01 ± 0.23	27.88 ± 0.28	21.65 ± 0.30
**pH**							
08:00 AM	7.70 ± 0.45–7.68 ± 0.11	8.25 ± 0.11	8.30 ± 0.06	8.39 ± 0.02	8.38 ± 0.01	8.36 ± 0.04	8.30 ± 0.01
12:00 PM	8.00 ± 0.45–8.20 ± 0.11	8.52 ± 0.10	8.53 ± 0.04	8.51 ± 0.03	8.51 ± 0.02	8.42 ± 0.01	8.35 ± 0.02
04:00 PM	8.10 ± 0.28–8.22 ± 0.23	8.44 ± 0.08	8.52 ± 0.04	8.54 ± 0.05	8.56 ± 0.03	8.47 ± 0.01	8.40 ± 0.02
12:00 AM	7.36 ± 0.76–7.20 ± 0.87	8.42 ± 0.06	8.43 ± 0.07	8.40 ± 0.01	8.44 ± 0.01	8.41 ± 0.01	8.32 ± 0.02
**Dissolved Oxygen (mg/L)**							
08:00 AM	1.76 ± 0.42–1.67 ± 0.42	3.13 ± 0.32	3.15 ± 0.42	3.14 ± 0.42	3.54 ± 0.36	3.70 ± 0.24	3.78 ± 0.22
12:00 PM	2.20 ± 0.42–2.21 ± 0.42	10.25 ± 1.33	10.23 ± 1.26	10.20 ± 1.24	8.74 ± 0.88	6.88 ± 0.28	5.91 ± 0.40
04:00 PM	2.23 ± 0.34–2.31 ± 0.57	10.67 ± 1.34	10.82 ± 1.32	10.85 ± 1.41	9.64 ± 1.03	7.84 ± 0.39	6.76 ± 0.50
12:00 AM	1.41 ± 0.45–1.34 ± 0.13	4.46 ± 0.36	4.44 ± 0.42	4.41 ± 0.45	4.54 ± 0.31	5.33 ± 0.27	5.13 ± 0.31

Both in control and raceways, fish were fed with commercial floating soy-based feed (Moisture: 10.17 ± 0.14%, Protein: 30.43 ± 0.28%, Fat: 4.77 ± 0.08%, Fiber: 4.21 ± 0.14%, Ash: 7.40 ± 0.34%, Starch: 23.00 ± 0.34%) (Supreme Aqua Feeds, Pvt. Ltd., Pakistan). Feeding rate varied according to water temperature and their body weight (at the rate of 2% of body weight in June and July, 1.5% of body weight from August till November). In control group, fish were fed by hand two times a day following the feeding table for tilapia given by Veverica and Janjua (2016) (2% of body weight). Use of auto feeders is not practice in traditional farming. However, in raceways, feeding was managed by auto feeders during day time. Feed delivery rate from auto feeders was adjusted according to quantity of feed ration over different months. Monthly weight check other than sampling was performed to adjust the feed ration for both groups over the grow out period. About a total of 200 fish from each raceway were randomly weighed on each weight check. However, monthly weight check could not be performed regularly in control ponds due to larger area and smaller stocking density. It was randomly done after almost two months and a few fish (n = 50) could be netted for weight check. Feeding was stopped after 28 November, 2019. First harvesting from raceways and control was carried out on November 30. The estimated biomass in each raceway and control ponds was adjusted monthly by subtracting mortalities from the number of fish stocked then multiplying that value by average fish weight to adjust the feeding rate.

Throughout the study, fish were morphologically (gills, abdomen and skin) monitored for any signs of disease. Moreover, they were morphologically examined for disease or any abnormality on each monthly weight check. Carcass of morts were examined for pathological and microbiological studies to identify the reason of mortality. Maximum and minimum values of water temperature (°C), pH and dissolved oxygen (DO) (mg/L) were measured both in raceways and control pond at two different depths (0.5 ft and 4 ft), at four different timings in 24 h (8:00 AM, 12:00 PM, 4:00 PM and 12:00 AM) on daily basis.

### 2.3 Sampling and analysis

Sampling protocol was followed after the approval of Animal Ethics Committee of Department of Zoology, Lahore College for Women University (Reference No: RERC/LCWU/Zoo-673–675). A total of 25 fish were randomly collected on monthly basis after every 30 days from June till November 2019 from each IPRS raceway. Large number of samples could not be collected as the site was commercial and funds were not sufficient to perform analysis of samples in larger number. Fish in raceways were not fed 24 h before each sampling time. Sampling (n = 20) from control ponds could be performed only once on November 30, 2019, when whole ponds were drained to harvest fish. Monthly sampling of fish from IPRS raceways was possible as fish were confined in small water area (220 m^3^) and in high stocking density.

Sampled fish were anesthetized by using 0.8 ml of clove oil (Sigma-Aldrich) per liter of water. Total body weigh nearest to ±0.1 g and total body length nearest to ±0.1 cm was measured. Blood samples were collected from caudal vein of fish and stored in EDTA coated tubes. Fish samples were stored at −4 °C until proximate analysis, determination of fatty acids and amino acids profiles were performed according to standard protocols of AOAC (2005). Fish blood samples were centrifuged (402*g* for 15 min at 5000 RPM) and plasma was stored at −4 °C until cortisol assay was performed. Levels of cortisol in plasma were measured by ELISA (Calbiotech, USA). Lower and upper limit of the test was 0–800 ng/ml at optical density of 450 nm. Coefficient of variation for intra assay and inter assay precision was 3.8% and 8.65%, respectively. Sensitivity of assay was 1.16 ng/ml while cross reactivity with testosterone, androstenedione, estradiol-17ᵝ and progesterone was <0.09%, <0.04%, <0.31% and <0.07%, respectively. All samples were run in replicates.

### 2.4 Growth parameters

At harvesting, total biomass was weighed for each cohort of fish in raceways and both control ponds. Condition factor, specific growth rate, hepatosomatic index, daily weight gain per fish, survival rate, FCR and daily gain in biomass were calculated by following formulae:

Condition factor = (total body weight/total body length^3^) × 100
Specific growth rate (%) = [(ln final weight − ln Initial weight)/time interval in days] × 100
Hepatosomatic index (HSI) *=* Weight of liver/total body weight × 100
Daily weight gain/Fish = [(total production/number of days of grow out period)]/total number of fish harvested
Survival rate = [(initial stocking density − number of morts)/initial stocking density] × 100
Feed conversion ratio (FCR) *=* Weight of feed consumed/(total final body weight of fish − initial total body weight of fish)
Total daily gain in biomass = Final biomass/number of days of grow out period

### 2.5 Statistical analysis

Mean ± SE of values of all parameters noted in two raceways and two control ponds at each point/month have been given. For comparisons between IPRS raceways and control groups, a *t*-test analysis of variance (ANOVA) was performed in SPSS17.0, followed by the least significant differences (LSD) test. A probability level of 0.05 was used to reject the null hypothesis.

## 3 Results

### 3.1 Water temperature, pH and dissolved oxygen (DO)

Water temperature and pH were found to be similar between raceways and control ponds from June till November, 2019 (Table 1). Water temperature in control and both raceways varied within the range of 33.95 ± 0.41–20.61 ± 0.26 °C during the grow out period. However, the levels of DO in raceways was higher as compared to those in control ponds due to aeration. Significant difference (*P* < 0.05) was observed between values of DO, measured at four different timings during 24 h in both groups (Table 1). In raceways, water temperature, pH and DO were measured at the depth of 0.5 ft and 4 ft were found to be insignificantly different (*P* > 0.05) when measured at four different timings during 24 h throughout the grow out period. Mean of data collected at two different depths have been given as mean of two values (at 0.5 ft and 4ft), given in Table 1.

### 3.2 Growth and production

Due to highs stocking density, total harvested biomass in both raceways (56.86 kg/m^3^ in raceway-1 and 57.81 kg/m^3^ in raceway-2) over grow out period of 171 days is larger than that obtained in both control ponds (average 0.38 kg/m^3^) (Table 2). Daily weight gain per fish in both control ponds and raceways was within the range of 4.59–4.62 g/day/fish/. Average final weight achieved in raceways and control groups was 769.50 ± 51.43 g and 787.50 ± 42.86 g, respectively. FCR in control ponds (1.24) is similar to that noted in both raceways (1.25). Survival rate was observed to be above 99% in both control ponds and raceways (Table 2).

**Table 2:** Summary of production and other parameters in traditional earthen pond (control) and IPRS raceways, culturing Tilapia (*Oreochromis niloticus*) for 171 days. Area of control pond was 6167 m^3^ while each raceway had water area of 220 m^3^.

#	Location	Stocking Density		Initial Biomass			Morts	Survival Rate	Harvested Biomass (kg)			Biomass Gained (kg)		Wt. Gain/Day/Fish (g)	FCR
		#	Fish/m^3^	Avg. I. Wt.(g)	Total Biomass (kg)	kg/m^3^	#	%	Avg. F. Wt. (g)	Total Biomass (kg)	kg/m^3^	Total Gain	Total Gain/Day		
1	Control-1	3000	0.49	32.00	96.00	0.015	16	99.45	790.00	2357.40	0.38	2261	13.22	4.62	1.24
2	Control-2	3000	0.49	32.00	96.00	0.015	25	99.17	785.00	2335.37	0.38	2239	13.10	4.59	1.24
3	Raceway-1	16,500	75.00	32.00	528.00	2.40	40	99.84	767.00	12,509.6	56.86	11981	70.07	4.44	1.25
4	Raceway-2	16,500	75.00	32.00	528.00	2.40	26	99.80	772.00	12,718.0	57.81	12190	71.30	4.51	1.25
Raceways		**33,000**	**–**		**1056**	**–**	**66**	**99.82**	**–**	**25,227**	**–**	**24,171**	**–**		**-**

#: Number, Avg. I. Wt.: Average initial weight; Avg. F. Wt.: Average final weight.

### 3.3 Condition factor (%), specific growth rate (SGR) (%) and hepatosomatic index (HSI) (%)

**Fig. 2:**
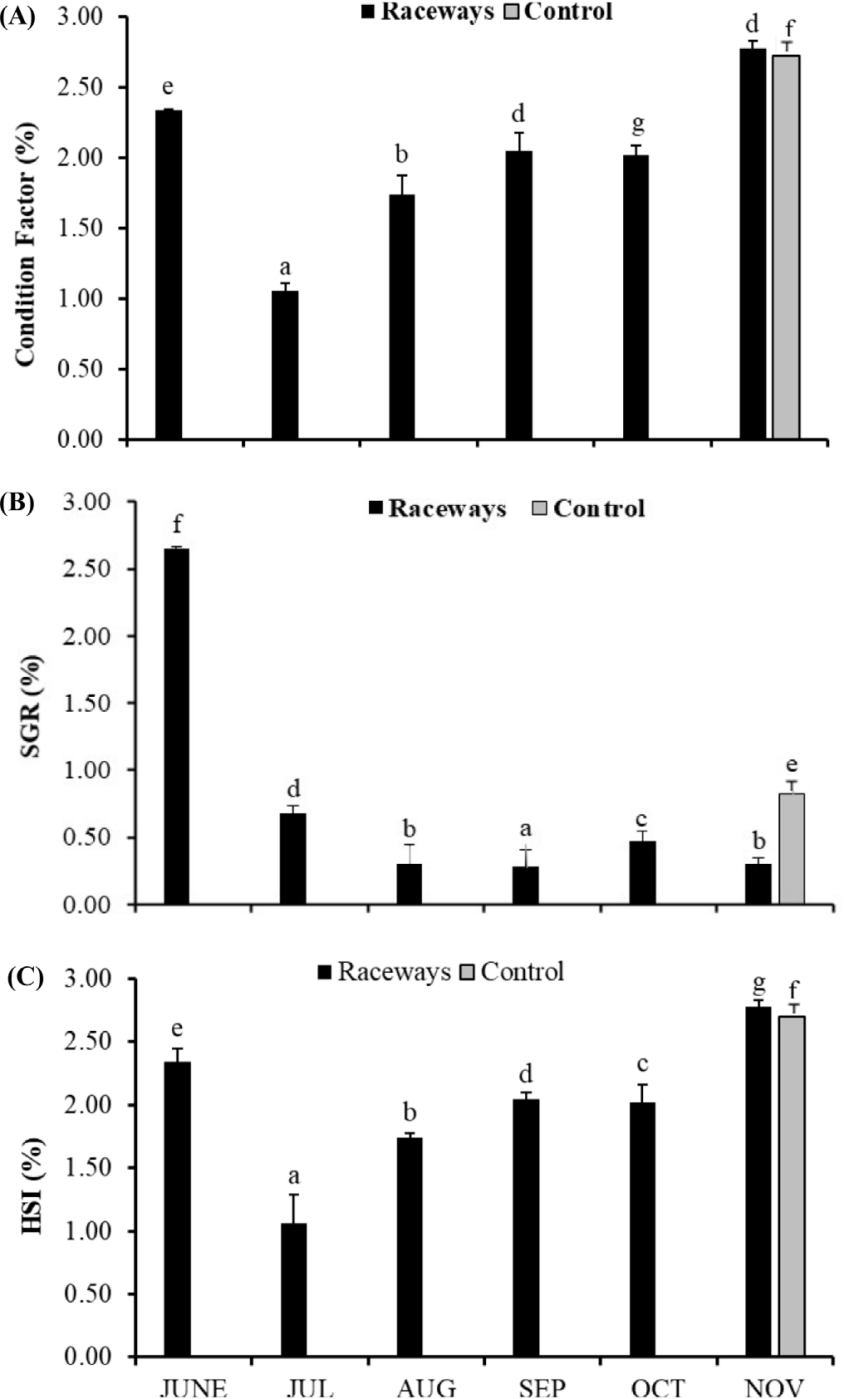
Profile (Mean ± SE) of Condition factor (A), Somatic growth rate (SGR) (B) and hepatosomatic index (HSI) (C) over the period of six months in raceways. Monthly samples could not be collected from control ponds. The given value of control represents the data collected in first week of December at the time of harvest. One way ANOVA computed difference between monthly values of proximate in raceways and control as well. Same letter indicates no significant difference among time points, but different letters indicate that values are significantly different at P < 0.05 for time points.

All parameters showed the significant difference (*P* < 0.05) between monthly values from raceways and control ponds (Fig. 2). In raceways, higher values of condition factor were noted in June and November (Fig. 2A). The highest value of SGR was observed in June and it remained within the range of 0.30 ± 0.14–0.68 ± 0.02% during remaining study period (Fig. 2B). The highest value of HSI was observed at end of the study in November (Fig. 2C). Other than November, HSI remained within the range of 1.06 ± 0.30–2.34 ± 0.11%.

### 3.4 Proximate analysis

A significant difference (*P* < 0.05) was noted between the values of crude protein, crude ash and crude lipids in muscle samples of control ponds and raceways (Table 3). Range of crude protein in both raceways and control ponds were observed within the range of 18.15 ± 1.34–20.00 ± 1.34%. Values of crude fats varied between 6.00 ± 1.48–7.26 ± 1.65% in both raceways and control ponds. Crude ash was found within the range of 1.38 ± 0.67–1.62 ± 1.03% in both groups.

**Table 3:** Proximate analysis (Mean ± SE) (%) of muscle samples collected from IPRS raceways and control pond. Monthly samples could not be collected from control ponds. The given value of control represents the data collected on November 30 at the time of harvest. One way ANOVA computed difference between monthly values of proximate in raceways and control as well. Same letter indicates no significant difference among time points, but different letters indicate that values are significantly different at P < 0.05 for time points.

#	Component	Control	IPRS Raceways					
			June	July	August	September	October	November
1	Moisture	72.90 ± 0.20**^b^**	74.00 ± 0.25**^f^**	73.21 ± 0.23**^c^**	74.00 ± 0.42**^ef^**	71.60 ± 0.20**^a^**	73.90 ± 0.34**^e^**	73.50 ± 0.33**^d^**
2	Crude Protein	19.00 ± 1.34**^d^**	18.60 ± 1.54**^c^**	18.15 ± 1.34**^a^**	18.46 ± 1.24**^b^**	20.00 ± 1.34**^e^**	18.60 ± 1.45**^c^**	18.63 ± 1.34**^c^**
3	Crude Ash	1.44 ± 0.65**^c^**	1.40 ± 0.77**^b^**	1.38 ± 0.67**^ab^**	1.36 ± 0.77**^a^**	1.62 ± 1.03**^e^**	1.51 ± 0.54**^d^**	1.40 ± 0.45**^b^**
4	Crude Fats	6.70 ± 0.97**^d^**	6.00 ± 1.48**^a^**	7.26 ± 1.65**^f^**	6.24 ± 1.54**^b^**	7.00 ± 0.56**^e^**	6.30 ± 0.67**^b^**	6.50 ± 1.04**^c^**

### 3.5 Profile of fatty acids

A total of 16 species of fatty acids were determined in fish muscle samples (Table 4) in all cohorts. All fatty acids showed significant difference (*P* < 0.05) between control and monthly values of fatty acids in both raceways. Total polyunsaturated fatty acids (PUFAs) in raceways were significantly higher than those in control (*P* < 0.05) especially α-linolenic acid, eicosapentanoic acid (EPA) and decosahexanoic acid (DHA). Highest levels of palmitic acid and oleic acid were observed among saturated fatty acids (SFAs) and monounsaturated fatty acids (MUFAs), respectively, both in control and raceways. Ratio of n-3/n-6 in both raceways were also significantly higher (*P* < 0.05) than control.

**Table 4:** Determination of saturated and unsaturated fatty acids (Mean ± SE) in total lipids extracted from muscle samples, collected from IPRS raceways and control. Values are expressed as percentage of total fatty acids. Monthly samples could not be collected from control ponds. The given value of control represents the data collected on November 30 at the time of harvest. One way ANOVA computed difference between monthly values of proximate in raceways and control as well. Same letter indicates no significant difference among time points, but different letters indicate that values are significantly different at P < 0.05 for time points.

**Saturated Fatty Acids (SFA)**									
**#**	**C: D**	**Common Name**	**Control**	**June**	**July**	**August**	**September**	**October**	**November**
1	C14:0	Myristic Acid	2.70 ± 0.87**^b^**	3.13 ± 0.42**^e^**	3.00 ± 0.35**^c^**	3.10 ± 0.45**^d^**	2.50 ± 0.63**^a^**	3.70 ± 0.45**^f^**	4.10 ± 0.78**^g^**
2	C15:0	Pentadecylic Acid	0.98 ± 0.06**^a^**	1.21 ± 0.56**^c^**	1.20 ± 0.55**^c^**	1.18 ± 0.46**^c^**	1.30 ± 0.03**^d^**	1.10 ± 0.34**^b^**	1.08 ± 0.25**^b^**
3	C16:0	Palmitic Acid	35.10 ± 1.23**^g^**	32.10 ± 1.24**^c^**	33.10 ± 1.18**^e^**	30.10 ± 1.24**^a^**	33.40 ± 1.03**^f^**	32.20 ± 1.45 **^d^**	31.30 ± 0.45**^b^**
4	C17:0	Margaric Acid	0.30 ± 0.06**^cd^**	0.31 ± 0.03**^cd^**	0.32 ± 0.05**^d^**	0.31 ± 0.05**^cd^**	0.21 ± 0.02**^a^**	0.28 ± 0.05**^b^**	0.30 ± 0.04 **^bc^**
5	C18:0	Stearic Acid	6.20 ± 0.56**^c^**	6.45 ± 0.64**^d^**	6.85 ± 0.37**^f^**	6.15 ± 0.67**^b^**	7.50 ± 0.45**^g^**	6.00 ± 0.65 **^a^**	6.50 ± 0.82 **^e^**
**Total SFA**			45.28 ± 2.34**^c^**	43.20 ± 2.32 **^a^**	44.47 ± 2.65**^a^**	40.86 ± 2.71**^a^**	40.86 ± 2.71**^a^**	43.28 ± 1.35**^b^**	43.28 ± 2.05**^b^**
**Monounsaturated Fatty Acids (MUFA)**									
1	C14:1n-5	Tetrasenoic Acid	0.23 ± 0.02**^a^**	0.32 ± 0.04**^c^**	0.25 ± 0.03**^a^**	0.22 ± 0.03**^a^**	0.50 ± 0.02**^d^**	0.30 ± 0.02 **^bc^**	0.29 ± 0.04**^b^**
2	C15:1n-5	Pentadecenoic Acid	0.50 ± 0.02**^de^**	0.48 ± 0.03**^cd^**	0.51 ± 0.02**^de^**	0.52 ± 0.04**^e^**	0.38 ± 0.01**^a^**	0.45 ± 0.06**^b^**	0.48 ± 0.02**^c^**
3	C16:1n-7	Palmitoleic Acid	9.40 ± 0.98**^a^**	11.78 ± 1.77**^c^**	12.61 ± 1.81**^e^**	11.60 ± 1.87**^b^**	12.00 ± 1.67**^d^**	13.80 ± 1.30**^g^**	13.30 ± 1.67**^f^**
4	C17:1n-10	Heptadecenoic Acid	1.51 ± 0.56**^bc^**	1.48 ± 0.53**^ab^**	1.52 ± 0.66**^c^**	1.50 ± 0.46**^bc^**	1.46 ± 0.54**^a^**	1.48 ± 0.16**^b^**	1.51 ± 0.51 **^bc^**
5	C18:1n-9	Oleic Acid	16.50 ± 1.56**^a^**	20.13 ± 1.30**^c^**	20.83 ± 1.12**^e^**	21.80 ± 1.03**^g^**	20.30 ± 1.92**^d^**	21.30 ± 1.55**^f^**	19.85 ± 1.78**^b^**
**Total MUFA**			28.14 ± 2.43 **^a^**	34.19 ± 3.32**^c^**	35.72 ± 2.54**^c^**	35.64 ± 3.41**^c^**	35.64 ± 3.41**^c^**	37.33 ± 2.10**^d^**	35.43 ± 1.64**^c^**
**Polyunsaturated Fatty Acids (PUFA)**									
1	C18:2n-6	Linoleic Acid	6.50 ± 0.43**^g^**	5.83 ± 0.43**^d^**	6.12 ± 0.32**^e^**	6.30 ± 0.23**^f^**	5.40 ± 0.57**^a^**	5.80 ± 0.67**^c^**	5.50 ± 0.54**^b^**
2	C20:2n-6	Eicosadienoic Acid	2.68 ± 0.42**^d^**	2.64 ± 014**^c^**	2.54 ± 0.42**^b^**	2.70 ± 0.46**^f^**	2.34 ± 0.78**^a^**	2.71 ± 0.46**^f^**	2.65 ± 0.32**^cd^**
3	C18:3n-3	α-linolenic Acid	7.50 ± 0.87**^a^**	12.46 ± 0.82**^b^**	12.89 ± 0.17**^e^**	12.50 ± 0.89**^c^**	12.50 ± 1.04**^c^**	12.80 ± 1.05 **^d^**	13.00 ± 1.05**^f^**
4	C20:5n-3	Eicosapentanoic Acid	4.80 ± 0.56**^a^**	6.56 ± 0.523**^d^**	6.12 ± 0.64**^b^**	6.70 ± 0.53**^e^**	6.50 ± 0.67**^c^**	6.80 ± 0.64**^f^**	7.60 ± 0.67**^g^**
5	C22:5n-3	Decosapentanoic Acid	5.50 ± 0.34**^a^**	7.67 ± 1.02**^f^**	7.34 ± 1.02**^d^**	7.50 ± 1.03**^e^**	8.20 ± 0.45**^g^**	7.30 ± 0.83**^c^**	7.00 ± 0.93**^b^**
6	C22:6n-3	Decosahexanoic Acid	3.80 ± 0.67**^a^**	4.34 ± 0.87**^d^**	4.56 ± 0.54**^e^**	4.20 ± 0.67**^c^**	4.70 ± 0.88**^f^**	4.10 ± 0.76**^b^**	5.35 ± 0.56**^g^**
**Total PUFA**			30.78 ± 1.45 **^a^**	39.50 ± 1.53**^b^**	39.57 ± 1.23**^b^**	39.90 ± 1.43**^b^**	39.90 ± 1.43**^b^**	39.51 ± 1.35**^b^**	41.10 ± 3.40**^c^**
**Total UFAs**			58.92 ± 1.94 **^a^**	73.69 ± 2.42**^c^**	75.29 ± 1.88**^c^**	75.54 ± 3.02**^c^**	75.54 ± 2.88**^c^**	76.84 ± 2.37**^d^**	76.53 ± 2.52**^d^**
**n-3/n-6**			2.35 ± 0.56 **^a^**	3.66 ± 0.72**^b^**	3.57 ± 0.45**^b^**	3.48 ± 0.75**^b^**	3.43 ± 0.67**^b^**	3.64 ± 0.76**^c^**	4.04 ± 1.02**^d^**

### 3.6 Profile of amino acids

A significant difference (*P* < 0.05) was noted between ten essential amino acids (EAA) and nine non essential amino acids (NEAA), determined in control and raceways (Table 5). Although statistically, these values showed significant difference however, quantitatively, no large difference was observed between six monthly mean values of raceways and control ponds. Leucine, lysine, threonine and arginine constituted the high EAA concentration were observed in all cohorts over the study period. Ornithine showed the lowest level in EAA. Among NEAA, Glutamic Acid was found to be the highest both in raceways and control group. TNEAA were noted be higher that TEAA throughout the study period. Total sulfur amino acids (TSAA) were determined to be within the range of 29.72 ± 0.25–32.23 ± 0.1 mg/gcp in all groups over study period. Values of total aromatic amino acids (TArAA) were found to be in range of 50.70 ± 1.35–56.00 ± 2.62 mg/gcp.

**Table 5:** Determination of essential and non-essential amino acids (Mean ± SE) in muscle samples collected from IPRS raceways. Values are expressed as mg of amino acid per g of crude protein (mg/gcp). Monthly samples could not be collected from control ponds. The given value of control represents the data collected on November 30 at the time of harvest. One way ANOVA computed difference between monthly values of proximate in raceways and control as well. Same letter indicates no significant difference among time points, but different letters indicate that values are significantly different at P < 0.05 for time points.

#	Essential Amino Acids (EAA)							
	Amino Acids	Control	June	July	August	September	October	November
1	Methionine	22.86 ± 1.54**^d^**	21.34 ± 1.45**^a^**	22.54 ± 1.51**^c^**	22.25 ± 1.56**^b^**	23.96 ± 1.67**^f^**	23.03 ± 1.87**^e^**	24.07 ± 1.16**^g^**
2	Threonine	38.43 ± 1.60**^e^**	36.84 ± 1.14**^a^**	37.14 ± 1.55**^c^**	37.04 ± 1.67**^b^**	36.86 ± 1.67**^a^**	38.06 ± 1.68**^d^**	39.42 ± 1.98**^f^**
3	Valine	37.00 ± 1.70**^e^**	37.56 ± 1.71**^g^**	36.92 ± 1.45**^d^**	37.32 ± 1.78**^f^**	36.01 ± 1.90**^b^**	36.24 ± 1.57**^c^**	35.96 ± 1.67 **^a^**
4	Isoleucine	34.57 ± 1.31**^d^**	34.00 ± 1.23**^c^**	33.98 ± 1.46**^c^**	33.80 ± 1.34**^b^**	35.02 ± 1.87**^e^**	32.87 ± 1.88**^a^**	35.55 ± 1.65**^f^**
5	Leucine	61.86 ± 1.21**^g^**	60.18 ± 1.12**^e^**	58.48 ± 1.23**^b^**	59.58 ± 1.01**^d^**	58.83 ± 1.45**^c^**	55.90 ± 1.45**^a^**	61.55 ± 1.93**^f^**
6	Phenylalanine	33.29 ± 1.66**^e^**	33.12 ± 1.45**^d^**	33.54 ± 1.76**^f^**	32.39 ± 1.86**^c^**	34.73 ± 1.65**^g^**	31.60 ± 1.01**^a^**	32.09 ± 1.67**^b^**
7	Histidine	22.14 ± 1.61**^d^**	22.35 ± 1.53**^e^**	21.84 ± 1.45**^c^**	21.55 ± 1.51**^b^**	22.40 ± 1.34**^f^**	20.79 ± 1.54**^a^**	22.68 ± 1.38**^g^**
8	Lysine	59.14 ± 1.11**^g^**	56.35 ± 1.14**^e^**	55.85 ± 1.10**^c^**	57.75 ± 1.20**^f^**	56.28 ± 1.99**^d^**	55.06 ± 1.89**^a^**	55.60 ± 1.02**^b^**
9	Arginine	49.57 ± 1.45**^d^**	49.00 ± 1.44**^c^**	48.56 ± 1.65**^b^**	48.31 ± 1.63**^a^**	51.46 ± 1.56**^e^**	49.02 ± 1.45**^c^**	52.97 ± 1.54**^f^**
10	Ornithine	2.14 ± 0.30**^a^**	2.34 ± 1.05**^c^**	2.23 ± 1.60**^b^**	2.25 ± 1.40**^b^**	2.13 ± 1.70**^a^**	2.39 ± 1.60**^d^**	2.63 ± 1.08 **^e^**
TEAA		361.0 ± 1.34	353.08 ± 1.32	351.08 ± 1.47	352.2 ± 1.50	357.7 ± 1.68	344.9 ± 1.60	362.5 ± 1.50
**Non-Essential Amino Acids (NEAA)**								
1	Cysteine	7.71 ± 1.04**^b^**	8.00 ± 1.04**^e^**	7.78 ± 1.05**^c^**	7.89 ± 1.06**^d^**	7.80 ± 1.07**^c^**	7.02 ± 1.03**^a^**	8.16 ± 1.04**^f^**
2	Aspartic Acid	63.57 ± 1.77**^d^**	62.56 ± 1.77**^b^**	63.86 ± 2.35**^e^**	63.10 ± 2.87**^c^**	60.53 ± 2.98**^a^**	65.45 ± 2.02**^f^**	67.36 ± 2.32**^g^**
3	Asparagine	56.00 ± 1.87**^d^**	56.73 ± 1.48**^e^**	55.67 ± 2.65**^c^**	55.63 ± 1.98**^b^**	60.53 ± 1.65**^f^**	54.21 ± 1.78**^a^**	61.83 ± 2.65**^g^**
4	Serine	36.57 ± 1.55**^g^**	34.63 ± 1.64**^d^**	35.13 ± 1.35**^e^**	36.34 ± 1.65**^f^**	31.76 ± 1.34**^b^**	30.76 ± 1.56**^a^**	33.06 ± 1.67**^c^**
5	Glutamic Acid	112.43 ± 2.72**^e^**	110.42 ± 2.37**^c^**	109.64 ± 2.77**^b^**	111.41 ± 2.67**^d^**	118.80 ± 2.52**^g^**	108.85 ± 2.54**^a^**	114.94 ± 2.34**^f^**
6	Glycine	55.14 ± 1.42**^g^**	53.65 ± 1.54**^d^**	52.89 ± 1.64**^c^**	54.65 ± 1.34**^f^**	52.74 ± 1.97**^b^**	51.12 ± 1.89**^a^**	53.80 ± 1.76 **^e^**
7	Alanine	58.86 ± 1.34**^f^**	57.42 ± 1.32**^d^**	55.65 ± 1.04**^c^**	58.45 ± 1.24**^e^**	54.72 ± 1.67**^a^**	55.06 ± 1.76**^b^**	60.03 ± 1.91**^g^**
8	Proline	39.00 ± 1.22**^b^**	40.00 ± 1.42**^f^**	39.55 ± 1.52**^d^**	39.15 ± 1.32**^c^**	39.69 ± 1.78**^e^**	38.20 ± 1.53**^a^**	41.08 ± 1.34**^g^**
9	Tyrosine	20.29 ± 1.75**^e^**	19.62 ± 1.75**^c^**	18.92 ± 1.56**^a^**	20.42 ± 1.75**^f^**	19.99 ± 1.78**^d^**	19.10 ± 1.81**^b^**	21.58 ± 1.65**^g^**
TNEAA		449.6 ± 1.63	443.03 ± 1.60	439.09 ± 1.77	447.4 ± 1.76	446.6 ± 1.86	429.8 ± 1.76	461.8 ± 1.85
TSAA		30.57 ± 1.29	29.34 ± 1.24	30.32 ± 1.28	30.14 ± 1.31	31.76 ± 1.37	30.06 ± 1.45	32.23 ± 1.10
TArAA		53.57 ± 1.1.70	52.74 ± 1.60	52.46 ± 1.66	52.82 ± 1.80	54.72 ± 1.71	50.70 ± 1.41	53.66 ± 1.66

TEAA: total Essential amino Acids, TNEAA: Total Non Essential Amino Acids, TSAA: Total Sulfur Amino Acids, TArAA: Total Aromatic Amino Acids.

### 3.7 Profile of cortisol

**Fig. 3:**
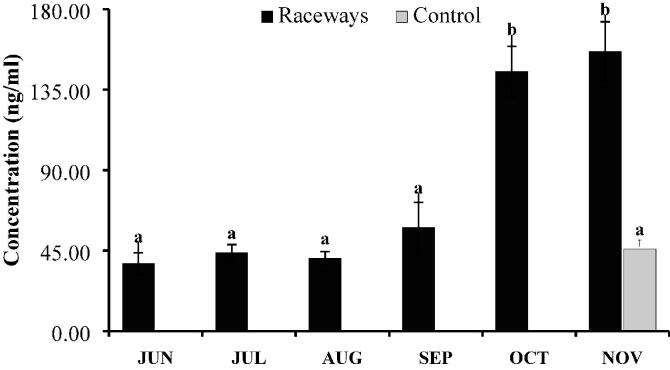
Concentration of cortisol (Mean ± SE) over the period of six months in raceways. Monthly samples could not be collected from control ponds. The given value of control represents the data collected in first week of December at the time of harvest. One way ANOVA computed difference between monthly values of proximate in raceways and control as well. Same letter indicates no significant difference among time points, but different letters indicate that values are significantly different at P < 0.05 for time points.

Levels of cortisol have been found significantly different (*P* < 0.05) between monthly values in raceways and control ponds (Fig. 3). However, no significant difference in concentration of cortisol was observed from June till September. Its level increased in October and remained high till end of study in November. In control, level of cortisol was lower (P > 0.05) as compared to that in raceways in November.

### 3.8 Economic feasibility

The total capital cost to construct three raceways in present study including pond modification, foundation was $22,222 ($1 = PKR 144) or $7407 per raceway (based on market rates in 2019). The total cost of equipment and machinery including aeration system, feeders, waste collection was $22,917 or $7639 per raceway excluding the cost of backup generator system ($3819). A total capital cost of $48,958 was required to build and operate the production system in present study. Total operational cost over the grow out period of 171 days was estimated to be $35,936. However, return on investment excluding the capital cost was 47.05 and breakeven point was achieved at 14,716 units in present study.

## 4 Discussion

In present study, total harvested biomass in two raceways was 25,227 kg (average 57.33 kg/m^3^) as compared to 4693 kg (0.38 kg/m^3^) in control earthen ponds of total ten acres area. Average production of any species in traditional farming in Pakistan is observed within the range of 1500–3500 kg per acre (4047 m^3^) or average 2600 kg/ha while using commercial feed and limited continual aeration in summer (PARC, 2013, Janjua, 2015). Usual stocking density of fish, particularly major carps and tilapia in traditional pond is 800–1000 fingerlings per acre in Pakistan. In present study, high production from two raceways (area of each raceway: 220 m^3^) indicates the potential opportunities of intensive aquaculture expansion by using IPRS technology, particularly when cage culture and RAS have proven not to be feasible in this country.

Comparison of this study with very few available previous studies performed in primary and modified forms of present IPRS system is difficult as pond area, number of raceways and area of raceway in these studies were not uniform. Moreover, considerable differences were present in design of IPRS, climate, duration of grow out period, initial stocking weight, average harvest fish size and types of cultured species between previous studies. Results achieved in present study have been found to be comparable with previous investigations. Wang et al. (2019) reported net yield of 22.15 kg/m^3^ of Tilapia from three raceways. Arana et al. (2018) observed the harvest density of 27–60 kg/m^3^ in IPRS while total final biomass was 47,140 kg from seven raceways. In China, where IPRS technology is in use since 2013, commercial production of Tilapia and Grass carp (*Ctenopharyngodon idella*) has been reported up to 136 kg/m^3^ and 100 kg/m^3^, respectively (Zhengreng, 2018, Pers. Commun.). Other than tilapia, Brown et al. (2011) reported the production of 7506 kg/ha of channel catfish and 13,034 kg/ha of hybrid catfish having the density range of 199 kg/m^3^ and 214.7 kg/m^3^ from seven raceways. Fullerton (2016) reported 33,531 kg of hybrid catfish from eight raceways. Conclusively, in present study, observed production per cubic meter from two raceways is comparable with previous studies performed in primary/different forms of IPRS, showing that water quality, pond and feed management were well maintained in present study.

Better growth and survival of fish in traditional earthen ponds has been observed in previous studies at smaller stocking density in comparison to that in present study in tilapia (Ronald et al., 2014, Rahman et al., 2016). Few studies have reported good growth and survival rate at high stocking density in different species in traditional earthen ponds e.g. Vundu catfish (*Heterobranchus longifilis*; Toko et al., 2007) and African catfish (*Clarias gariepinus*; Toko et al., 2007). However, high stocking density in above mentioned studies was still lower as compared to present study. Most of the studies on effects of high stocking densities have been performed in tanks and reported lower growth rate and survival in high stocking density cohorts in different species (Irwin et al., 1999, Al-Harbi and Siddiqui, 2000, Jha and Barat, 2005, Schram et al., 2006, Szkudlarek and Zakęś, 2007).

In present study, overall survival of tilapia was observed to be above 99% which is better than those observed in previous studies in primary/modified forms of IPRS. Total mortality of almost 1% was due to sudden spell of cold during the last two weeks of grow out period. In IPRS, survival rate of tilapia has been reported to be 86.23% (Wang et al., 2019), 43–92% (Arana et al., 2018), 92% (Wang et al., 2018) and 94.1% (Masser, 2017). Channel catfish showed survival rates of 47–69.1% (Fullerton, 2016), 77.2% (Masser, 2016), 83.7% (Brown et al., 2010, Brown et al., 2011), 98% (Masser and Lazur, 1997) 58.9% (Martin,1997), 85.6% (Wilcox, 1998), 54.4–83.6% (Hawcroft, 1994), 67.5–81.4% (Bernárdez, 1995) in different forms of IPRS system. Survival rate of hybrid channel catfish was 89.1% in IPRS Raceways (Brown et al., 2010, Brown et al., 2011). Comparatively, present study showed the highest survival rate of tilapia in IPRS at the biomass density of 57.33 kg/m^3^ in raceways, suggesting that well maintained water quality and husbandry conditions supported survival of fish despite high stocking density in confined area. However, similar rate was also observed in earthen ponds where lower stocking density ensured the survival of fish.

FCR is extremely important in economic feasibility as commercial fish feed costs major part of operational expenses. In present study, average FCR observed for tilapia was 1.25 in both raceways. FCR noted in present study is similar to that reported in previous studies performed in IPRS or its primary forms. The average FCR for channel catfish in IPRS system has been observed to be 1.59–2.14 in different raceways (Fullerton, 2016), 1.74 (Brown et al., 2010, Brown et al., 2011) 1.41–1.75 (Bernárdez, 1995), 1.45–1.57 (Wilcox, 1998) and 1.85–1.95 (Hawcroft, 1994). Arana et al. (2018) observed the FCR of 1.36 from tilapia cultured in IPRS raceways. In hybrid channel catfish, it was noted to be 1.36 ((Brown et al., 2010, Brown et al., 2011). In present study, it can be inferred that total feed given in raceways was well consumed due to efficient feeding management and excellent fish husbandry conditions. In Pakistan, FCR is observed within the range of 4–8 in traditional earthen ponds (Janjua, 2015) which shows that FCR in tilapia improves with increase in stocking density if pond and feed management are excellent as observed in present study. Previous studies reported the negative impact of high stocking density on growth rate and production of Tilapia in traditional, cage culture and biofloc system (Abou et al., 2007, Gibtan et al., 2008, Chattopadhyay et al., 2013, Garcia et al., 2013, Telli et al., 2014, Moniruzzaman et al., 2015; Manduca et al., 2020). It shows the possible advantage of IPRS technology over other culture methods for tilapia production.

In present study, average growth rate was observed to be 4.67 g/fish/day, and which has been found higher than previously mentioned studies. In channel catfish, growth rates ranged from 1.43 to 1.78 g/fish/day (Fullerton, 2016), 1.1 to 1.8 g/fish/day (Brown et al., 2010, Brown et al., 2011), 2.50 g/fish/day (Martin, 1997), 3.50 g/fish/day (Bernárdez, 1995) and 1.00–2.50 g/fish/day (Hawcroft, 1994), 2.63–2.77 and 2.89 g/fish/day were observed in primary form of IPRS and cages, respectively (Wilcox, 1998). In hybrid catfish, growth rate was reported to be 1.60 to 2.20 g/fish/day (Brown et al., 2010, Brown et al., 2011). In tilapia, 2.60 and 3.07% gain/day was observed in IPRS (Arana et al., 2018) while Wang et al. (2019) reported decrease in growth rate in raceways. Higher growth rate in present study as compared to previous reports, showed that fish were fed to satiation, however, trade off impacts of feeding to satiation such as impairment in water quality and high FCR (Li and Lovell, 1992) were not observed at any stage in present study due to excellent husbandry conditions maintained throughout the grow out period.

Water quality was well maintained in the present study due to rich aeration, water circulation in whole system and removal of most of solid waste from raceways. At a water temperature of 26 °C, freshwater at 50% oxygen saturation contains about 4.00 mg/L of dissolved oxygen (Boyd et al., 2011) and it should not decline below 3.00 mg/L in raceways. In present study, average daily DO in raceways never dropped lower than 5.00 mg/L throughout the grow out period. Similar concentrations of DO at water inflow and outflow (22 m away from inflow) show the rich aeration and good mixing of water in IPRS raceways as previously reported by Brown et al. (2011). These high levels of DO, maintained in present study was the major reason of average 99% survival rate and better growth rate under high stocking density conditions. Evans et al., 2003, Boyd et al., 2011, Abdel-Tawwab et al., 2015 reported the adverse effects of low DO caused by high stocking density on tilapia growth, total production, survival, feed utilization, and innate immunity. Previous studies have reported the similar results in different species (Ellis et al., 2002, Welker et al., 2018, Welker et al., 2019). However, no such negative effect of high stocking density was observed in present study in case of survival, growth rate and FCR. Similar to DO, pH also remained within the recommended pH value for aquaculture i.e., 6.5–9.0 (Boyd et al., 1978) and suitable range for tilapia i.e. 5.5–9.0 (Rebouças et al., 2016).

Aquaculture products should meet the requirements of food regulations, commercial specifications and standards of World Health Organization (WHO) for human nutrition. High stocking density also reduces flesh quality (Refaey et al., 2018) other than growth, immune response and disease resistance in Tilapia (Telli et al., 2014, Manduca et al., 2020). However, in present study, careful assessment of protein and lipids quality was performed. Although fat content reported in different studies is highly variable depending upon the species, season, size, reproduction status, feed, density and environmental conditions (Taşbozan and Gökçe, 2017), fish in present study can be classified as medium fat fish (5–10% fat content by weight, Bennion, 1980) as previously reported (Memon et al., 2010) in Pakistan. Higher levels of total PUFA and important α‐linolenic acid, EPA and DHA in raceways supports that IPRS enhanced the nutritional value of tilapia muscle without being affected by high stocking density and seasonal changes. n-3/n-6 ratio in present study, is also within the recommended range of 1.6–2.0 by Henderson and Tocher (1987) in freshwater species. Similar results but comparatively low fractions of EPA and DHA were reported by Yuan et al. (2019) in Largemouth bass cultured in raceways. Levels of these fatty acids were also found to be higher than those reported in eight wild (Memon et al., 2010) and three farmed species in Pakistan (Khan et al., 2015). Higher proportion of unsaturated fatty acids than SFAs and acceptable range for total PUFA (n-6 and n-3 fatty acids) in present study are also an evidence that fish grown in IPRS are nutritionally up to Food and Agriculture Organization of The United Nations (FAO) standards of fats and fatty acids required in adult human nutrition if consumed in recommended quantity (FAO, 2010).

Protein content in present study despite of stress caused by high stocking density and seasonal changes was found to be comparable to five different tilapia species (Taşbozan et al., 2013), eight different freshwater species in Pakistan (Memon et al., 2010), broiler breast meat (Bostami et al., 2017), lamb and turkey meat (Cobos and Díaz, 2014). Although Manduca et al. (2020) reported reduction of crude protein in carcasses at the highest densities in tilapia in biofloc system, however high stocking density did not affect the protein content of tilapia in present study. Monthly profile of TEAA, NEAA, TSAA and TArAA in raceways were similar to those in control, showing that tilapia in high stocking density performs more efficiently in raceways than control having low stocking density. Contents of TEAA in present study are also comparable to previous studies performed in wild or traditional fish culture including tilapia (Adeyeye and Adamu, 2005, Adeyeye, 2009, Mohanty et al., 2014, Taşbozan and Gökçe, 2017). Levels of TSAA (methionine and cysteine) in both raceways are close to the recommended value of 22 mg/gcp for amino acids requirements in adult human nutrition (WHO/FAO/UNU, 2002). Although their occurrence in proteins is less abundant than other amino acids, they are metabolically important to the extent that their relative requirement for maintenance is probably higher than that for human growth. Threonine which is nutritionally rate limiting amino acid in body maintenance requirement has been higher in present study than human adult’s requirement (WHO/FAO/UNU, 2002). Leucine, lysine and valine, involved in muscle protein synthesis and other immunity and physiological maintenance were also up to recommended intake levels of adult humans which is 59, 45 and 39 mg/gcp, respectively (WHO/FAO/UNU, 2002). Similar to essential amino acids, adequate amounts of non-essential amino acids must be provided for effective utilization of EAA, as well as the synthesis of other physiologically important nitrogen-containing compounds, such as purines and pyrimidines, glutathione and creatine. Present study shows that tilapia grown under stocking conditions of IPRS is nutritionally good source of dispensable amino acids as well.

High stocking density is chronic stressor in aquaculture due to deteriorating water quality, abnormal social behavior such as social hierarchy, reduced food consumption and altered metabolic and hematological conditions (Fazio et al., 2014). Intensity of stress can be assessed by changes in blood nutrient, corticosteroid hormone and catecholamine levels may be used to reflect the responses to stressors (Bolasina et al., 2006, Saurabh and Sahoo, 2008). In present study, cortisol level was noted to be significantly higher in last two months of study (October and November) as compared to control ponds. This finding agrees with previous studies (Lupatsch et al., 2010, Varela et al., 2010, Yarahmadi et al., 2016, Fatima et al., 2018). However interestingly, this higher level of cortisol in last two months did not suppress growth, compromised immunity, caused diseases or mortality in raceways which can be attributed to better aeration, water quality, feeding management and reduced swimming of fish confined in raceways.

## 5 Conclusion

It can be suggested that negative effects of high stocking density were not observed in tilapia when culture using IPRS technology. Study showed that average biomass density of 57.33 kg/m^3^ of tilapia can be successfully achieved with excellent growth rate, survival rate, quality of protein and lipids contents without compromising the immunity and disease resistance of fish while using the IPRS technology. Therefore, targeted density per cubic meter can be further increased up to 75–100 kg/m^3^ in future. It can also be concluded that initial stocking weight higher than 30–32 g may result in better growth rate and production in raceways. Growth and survival rate of tilapia observed in present study has been found higher than previous studies on similar species in traditional earthen ponds and tanks (Al-Harbi and Siddiqui, 2000; Ronald et al., 2014; Rahman et al., 2016). However, there are vast future opportunities to modify the design and building material to make IPRS technology economically more feasible for medium scale farmers. Different species at higher stocking densities can also be studied in grow out phase with assessment of their immune and stress response. In present study, three raceways were built in 7 acres water area (2.84 ha). According to Chappell (2017), two raceways can be built per hectare if pond’s depth is 2 m. Following this rule, a total of six raceways could be built in 7 acre water area. Therefore, projected harvested biomass can be increased up to 75–150 kg/m^3^.

## Funding

This project was funded by Higher education Commission of Pakistan under Technology Development Fund Program (Project No: TDF03-132).

## Declaration of Competing Interest

The authors declare that they have no known competing financial interests or personal relationships that could have appeared to influence the work reported in this paper.
